# Association of single nucleotide polymorphisms (SNPs) with gastric cancer susceptibility and prognosis in population in Wuwei, Gansu, China

**DOI:** 10.1186/s12957-022-02663-6

**Published:** 2022-06-11

**Authors:** Ping Fan, Zhiyi Zhang, Linzhi Lu, Xingcai Guo, Zhicheng Hao, Xinghua Wang, Yancheng Ye

**Affiliations:** 1 Department of Pathology, Gansu Wuwei Tumor Hospital, Wuwei, 730000 Gansu China; 2 Department of Gastroenterology, Gansu Wuwei Tumor Hospital, Wuwei, 730000 Gansu China; 3 Biochip Center, Gansu Wuwei Tumor Hospital, Wuwei, 730000 Gansu China; 4 Biobank, Gansu Wuwei Tumor Hospital, Wuwei, 730000 Gansu China; 5 Department of Pharmacy, Gansu Wuwei Tumor Hospital, Wuwei, 730000 Gansu China

**Keywords:** Gastric cancer, Single nucleotide polymorphisms (SNP), Cancer susceptibility, rs4823921, Wuwei

## Abstract

**Background:**

Gastric cancer (GC) is the sixth most common cancer. China is one of the most frequent GC occurred countries, and Wuwei, Gansu, is one of the highest incidence area in China. Possible biomarkers of GC susceptibility and prognosis among the population in Wuwei are urgently needed.

**Methods:**

All participants in this study were recruited from the Wuwei Cancer Hospital in Gansu, including 303 patients diagnosed with GC and 200 non-cancer controls. DNA was extracted for further single nucleotide polymorphisms (SNP) genotyping. All SNPs were firstly screened by additive logistic regression model then selected SNPs were subjected to univariate Cox regression analysis and multivariate Cox regression analysis for their associations with GC occurrence.

**Results:**

The results showed that 31 SNPs were significantly related to the incidence of GC in Wuwei, Gansu, China. Genotype rs4823921 was significantly related to the overall survival of GC patients and AC/AA genotype of rs4823921 polymorphism was significantly associated with an increased risk of GC in Wuwei population.

**Conclusions:**

Thirty-one SNPs were significantly related to the incidence of GC in Wuwei and rs4823921 genotype AC/AA was significantly associated with poor prognosis of GC patients in Wuwei, Gansu.

**Supplementary Information:**

The online version contains supplementary material available at 10.1186/s12957-022-02663-6.

## Background

As a prevalent malignant tumor, gastric cancer (GC) is the fifth most common cancer and the third leading cause of cancer-related death around the world [1]. Despite the decreasing incidence globally in past years, GC incidence in Eastern Asian is still high [2]. As GC patients often show few typical symptoms at an early stage, the best opportunity for optimal treatment is unfortunately missed [3]. Thus, GC is usually found until it progresses to advanced stages in most cases [4]. Despite with the development of surgical techniques and chemotherapy, the 5-year survival rate of early GC has been improved to reach > 95% [5]. Whereas, most cases are usually diagnosed at an advanced stage, which makes GC become a problem with 5-year overall survival of less than 25% [6, 7]. Accordingly, the early detection of GC is crucial to improve the prognosis and reduce the mortality of GC patients.

Although GC is a public health problem worldwide, many studies have shown that it has a higher incidence and prevalence rate in Asian/Pacific regions, which indicates that region and race may be associated with the elevated risk of GC [6, 8, 9]. Among all GC cases occurred, about 75% occurred in Asia, and China is one of the most frequent countries, which accounts for 42.6% of the global incidence [9–11]. In China, Wuwei, Gansu, is one of the highest GC incidence’s area [12]. This may be related to various complex reasons, such as the preference for pickled foods [13], high helicobacter pylori infection rate [14], genetic factors [15], and so on. Researches have demonstrated that single nucleotide polymorphisms (SNP) sites play an important role in the development of many cancers [16–18]. SNP differences between different races and regions have different effects in cancer susceptibility, progression and prognosis [8, 16, 17, 19, 20]. A meta-analysis revealed that 72 polymorphisms in the codon of P53 gene may be associated with GC in Asians [17]. A GWAS (genome-wide association study) study showed that two SNP mutations in PSCA gene were associated with increasing risk of diffuse GC in Korean and Japanese populations [21]. At present, most studies about hereditary gastric cancer focused on germline mutations of tumor suppressor genes CDH1 (E-cadherin) [22–24]. But there are few studies on biomarkers of GC susceptibility and prognosis among the population in Wuwei. Therefore, it will be of great significance for local GC prevention if we could screen out the differential SNPs based on obtained GC patients information in Wuwei.

Herein, we aimed to screen out GC susceptibility mutation sites related to population in Wuwei through SNP chip, in order to provide more reference information for the early detection of GC in Wuwei.

## Methods

### Study population

This study recruited GC patients from the Wuwei Cancer Hospital in Gansu, China. There are 303 patients diagnosed with GC and 200 non-cancer controls, among which 97 patients have complete survival information. All GC patients were confirmed by histopathology and diagnosed from 2014 to 2019. The detailed histopathology and pathological TNM information, and the clinical information were shown in Table S1.

This study has been approved by the local hospital ethics committee (ethic code: 2019-Ethical Review-02). All participants recruited had provided written informed consent. Each participant received a standardized questionnaire survey, which collected age, gender, family history, pathological diagnosis, and other information. The clinical data and demographic characteristics of GC patients and controls have been summarized in Table 1.

**Table 1:** Clinical and demographic characteristics of cases and controls

Variables		Case (*N* = 303)	Control (*N* = 200)	*P*
Age	Mean	59	55	0.7079
	Range	30–79	42–67	
Gender	Male	223 (73.6%)	99(49.5%)	5e−14
	Female	59 (19.47%)	101(50.5%)	
	Unknown	21 (6.93%)	0	
Family history of cancer	YES	33 (10.89%)	–	
	NO	270(89.11%)	–	
Serum CEA	Mean	21.44	–	
Serum CA199	Mean	22.87	–	

*P* value was calculated with chi-square test

### Study subject inclusion and exclusion

Basing on our main researching purpose regarding GC susceptibility in Wuwei, the following inclusion criteria was adopted: (1) all research subjects voluntarily participated in the study and signed the informed consent. (2) The participants were treated in Wuwei Cancer Hospital in Gansu, China (01 January 2014–31 December 2019) and were pathologically diagnosed with GC/adenocarcinoma of esophagogastric junction. (3) The research subjects are permanent resident with Wuwei household registration. (4) Adult participants (age ≥ 18). (5) Healthy controls had free tumor history. The following exclusion criteria was adopted: (1) patients with non-adenocarcinoma of stomach or gastroesophageal junction (such as squamous cell carcinoma, undifferentiated carcinoma, neuroendocrine carcinoma, and lymphoma). (2) Patients who had previously received systemic chemotherapy for locally advanced unresectable or metastatic GC/adenocarcinoma of esophagogastric junction. (3) Patients suffering from other malignant tumors during past 3 years or at present. (4) Subjects with any other conditions unsuitable for participating in this clinical study.

### Sample collection and DNA extraction

For GC tissue DNA extraction, 15–25 mg tissue from each sample was taken to be cut into pieces. The Biospin DNA Extraction Kit (BSC04S1, Bioer Technology, Hangzhou, China) was used for further tissue DNA extraction, and all steps were strictly in accordance with the instructions of the kit. The DNA samples were stored at − 20 °C. For peripheral venous blood DNA extraction, 5 ml blood was taken from each specimen for further DNA extraction. The TIANamp Genomic DNA Kit (DP304, Qiagen Inc., Valencia, CA, USA) was utilized for blood DNA extraction, and the operating steps strictly followed the instructions of the kit.

### SNP genotyping

The Axiom® genotyping chip was conducted by Beijing Bode Biotechnology Co., Ltd., to process the sample genotyping. Then, 200 ng DNA from each sample was taken as DNA sample. After the whole genome amplified, the genomic DNA was randomly fragmented in segments between 25 and 125 bp. DNA fragments were then resuspended and hybridized with Axiom whole genome array plates.

The hybridization product was strictly washed with water to remove non-specific background and retain specific binding. Each SNP was identified by the coupling reaction on the chip surface. When the coupling reaction completed, the chip would finish the steps including staining and washing on GeneTitan multi-channel automated chip workstation, and finally the results were scanned and output.

### Selection of SNPs

The software PLINK 1.09 was used to perform quality control on the genotyping results, the samples with genotyping call rates < 95% were removed. Moreover, SNPs with too small minor allele frequency had little contribution, which should be filtered out. According to the Hardy-Weinberg genetic equilibrium testing, the gene frequencies were substituted to obtain genotype equilibrium frequencies, and then multiplied by the total population to obtain the estimations (*e*). Comparing observations (*O*) and estimations (e), *χ*^2^ test was conducted, the SNPs with Hardy-Weinberg equilibrium *P* value over controls of < 1 × 10^−5^ and linkage disequilibrium (LD) *r*^2^ ≥ 0.8 were also removed. Then, pairwise identity by state (IBS) potential genetic kinship checks were conducted on all successfully genotyped samples. On the identification of a first- or second-degree relative pair, we removed one of the two related individuals (the sample with the lower call rate was removed). The remaining samples were then evaluated for population outliers and stratification using principal component analysis (PCA)-based methods.

### Survival analysis

Overall survival (OS) was defined as the date from the diagnosis to death due to any reason. Follow-up information was obtained through telephone or outpatient data. The follow-up time was 6–61 months, and the median follow-up time was 38 months. Univariate Cox regression analysis was used to screen out the factors related to the OS of GC. The independent prognostic factors were determined by multivariate Cox proportional hazard model.

### Statistical analysis

Different groups of demographic variables were analyzed using chi-square test or Fisher’s exact test. The additive logistic regression model was used to estimate the correlation between SNPs and GC, odds ratio (OR) and 95% confidence intervals (CIs) were then obtained and *P* value was adjusted by FDR. The genotypes and alleles of different groups were compared using chi-square test or Fisher’s exact test and then checked whether their distributions were statistically different. Statistical significance was accepted for *P* < 0.05. All statistical analysis of data used R 3.6.2.

## Results

### Association of SNPs with gastric cancer susceptibility

**Fig. 1:**
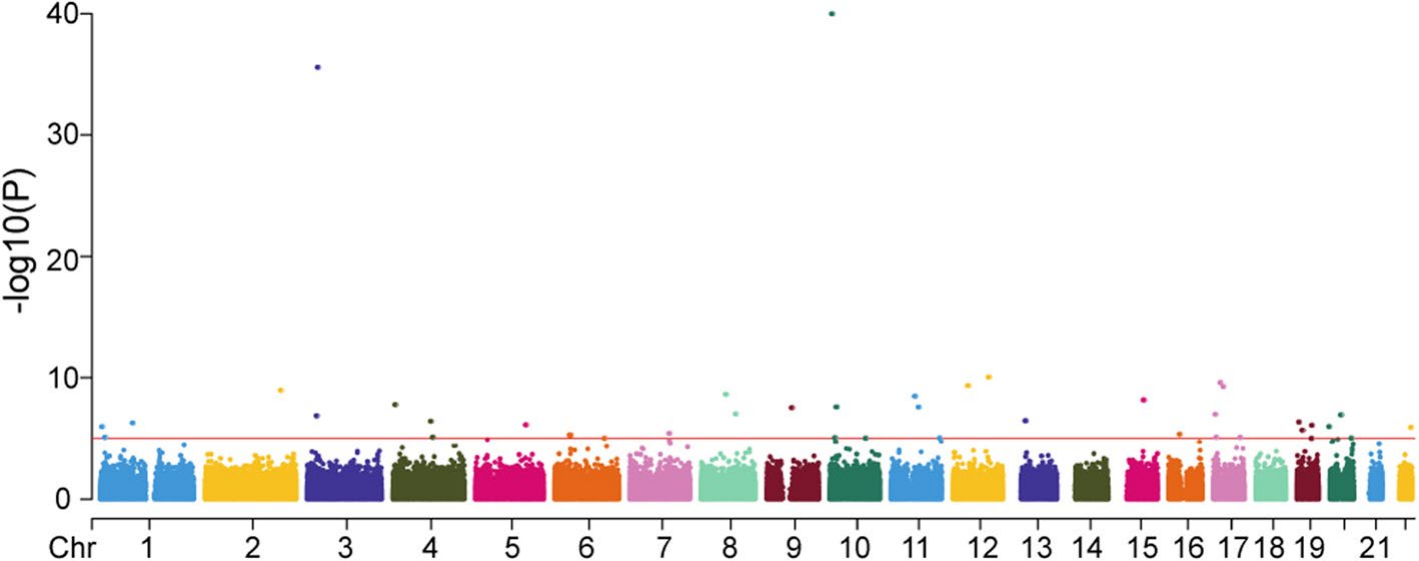
Manhattan plot of all SNP sites. The horizontal axis represents the chromosomes, and the vertical axis is −log10(*P* value). The higher position means the smaller *P* value. The red line is *P* value = 10^−5^

After quality control, we retained 482 samples (case 289, control 193) and 300,219 SNPs for association analysis. Through the additive logistic regression model, 42 SNPs were found to be significantly related to the onset of GC (*P* value < 1e−5) (Fig. 1). After FDR adjustment, 31 SNPs were related to the onset of GC (PFDR < 0.05). The gene frequency of GC risk related SNP loci (top 10) were listed in Table 2, and the genotype distribution of GC risk related SNP loci (top 10) were listed in Table 3. The distribution of gene frequency and genotype of all 31 loci were shown in Table S2 and Table S3.

**Table 2:** Alleles distribution of the top 10 significant SNPs related to gastric cancer risk

SNP ID	SNP position	Gene	Region	Allele	Case		Control		*P*_FDR_	OR (95%CI)
					*N*	%	*N*	%		
rs421490	chr1:8215224	LINC01714	Downstream	G	137	25.00	47	12.30	1.21E−02	2.38 (1.65–3.41)
				A	411	75.00	335	87.70		
rs786906	chr1:88805891	PKN2	Exonic	C	214	37.68	201	52.07	7.01E−03	0.56 (0.42–0.723)
				T	354	62.32	185	47.93		
rs200612063	chr2:203440845	RAPH1	Exonic	G	86	16.10	6	1.58	4.60E−05	11.97 (5.17–27.69)
				A	448	83.90	374	98.42		
rs141620966	chr3:27315885	NEK10	Intronic	C	86	16.10	2	0.52	2.26E−03	36.67 (8.96–150)
				T	448	83.90	382	99.48		
rs7640543	chr3:30420911	RBMS3, LINC01985	Intergenic	A	299	0.11	53.78	40.00	3.89E−31	9.54 (6.60–13.79)
				G	257	46.22	328	89.13		
rs28698945	chr4:8850597	HMX1	Intronic	G	111	20.48	26	6.81	4.38E−04	3.53 (2.25–5.528)
				A	431	79.52	356	93.19		
rs146971769	chr4:102980615	SLC9B1	Intronic	C	82	15.02	2	0.54	5.55E−03	32.69 (7.98–133.8)
				A	464	84.98	370	99.46		
rs78326603	chr5:135862851	SLC25A48	Intronic	C	76	13.82	2	0.53	9.74E−03	30.3 (7.39–124.2)
				T	474	86.18	378	99.47		
rs713383	chr6:42272631	TRERF1	Intronic	A	179	31.63	177	46.09	4.98E−02	0.54 (0.413–0.71)
				G	387	68.37	207	53.91		
rs9463078	chr6:45213603	SUPT3H	Intronic	G	174	31.64	170	45.45	4.98E−02	0.56 (0.423–0.73)
				A	376	68.36	204	54.55

**Table 3:** Genotype distribution of SNPs significantly related to gastric cancer risk

SNP	Allele	Group	AA	AB	BB	*P*
rs421490	G/A	Case	8	121	145	1.24E−07
		Control	4	39	148	
rs786906	C/T	Case	2	210	72	2.10E−22
		Control	56	89	48	
rs200612063	G/A	Case	0	86	181	2.09E−18
		Control	1	4	185	
rs141620966	C/T	Case	0	86	181	6.88E−21
		Control	0	2	190	
rs7640543	A/G	Case	35	229	14	8.55E−66
		Control	1	38	145	
rs28698945	G/A	Case	7	97	167	3.13E−09
		Control	0	26	165	
rs146971769	C/A	Case	0	82	191	1.36E−18
		Control	0	2	184	
rs78326603	C/T	Case	0	76	199	2.69E−17
		Control	0	2	188	
rs713383	A/G	Case	25	129	129	1.78E−05
		Control	36	105	51	
rs9463078	G/A	Case	3	168	104	3.54E−15
		Control	44	82	61	

### SNPs related to the prognosis of gastric cancer

**Fig. 2:**
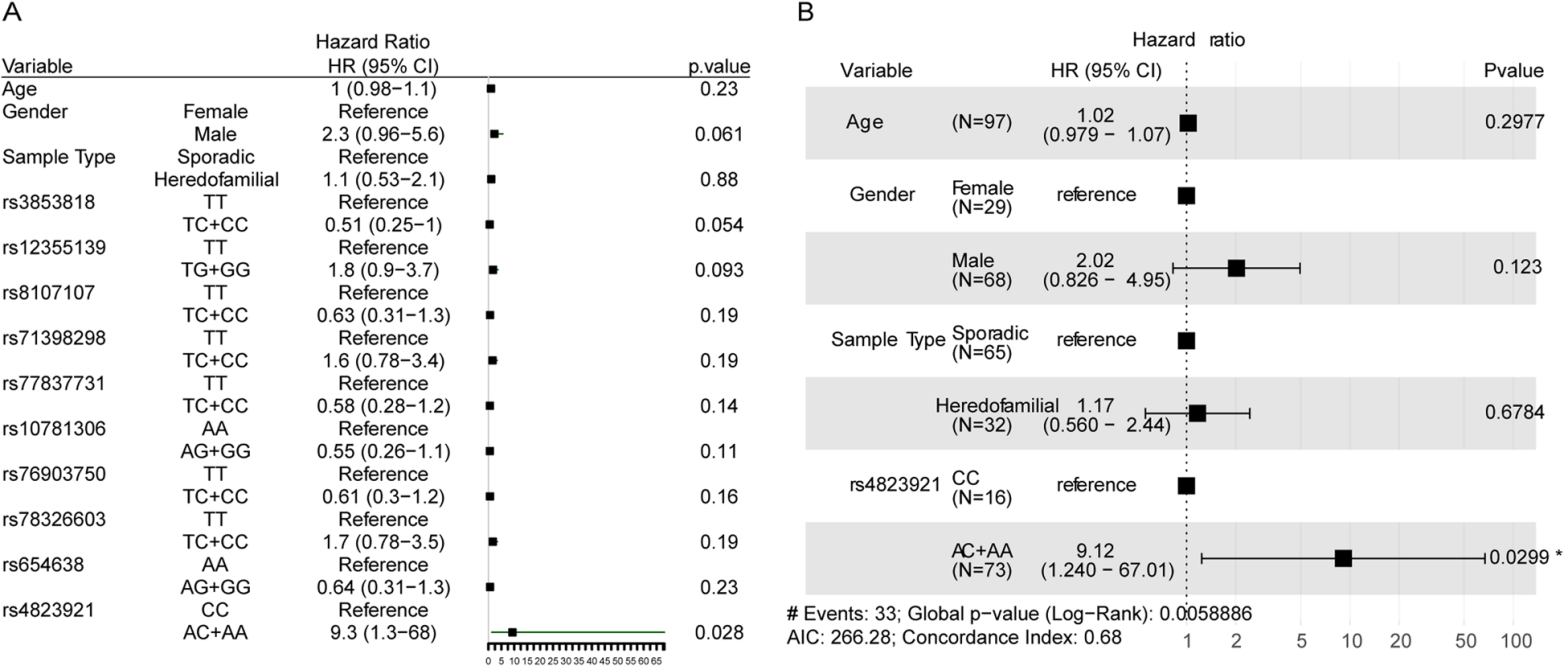
SNPs related to the prognosis of gastric cancer. **A** Univariate Cox regression analysis forest plot. **B** Multivariate Cox regression analysis forest plot. Compared with reference samples, samples with a hazard ratio greater than 1 have the higher risk of death, and samples with a hazard ratio less than 1 have the lower risk of death

We performed univariate Cox regression analysis on 97 gastric cancer patients with complete survival information, setting age, gender, sample type (distributed samples and family genetic samples), and 31 SNP loci as variable. Some of loci are shown in Fig. 2A and all results of 31 SNP loci were listed in Table S4. The results showed that only rs4823921 was significantly related to the overall survival rate of gastric cancer. Taking genotype CC as a reference, patients with genotype AC or AA had relatively poor prognosis (HR 9.3 (1.3−68), *P* = 0.028).

In order to confirm whether genotype of rs4823921 can be used as an independent prognostic indicator to predict the prognosis of gastric cancer patients, we performed multivariate Cox regression analysis setting age, gender, sample type (distributed samples and family genetic samples), and rs4823921 as variable, results showed in Fig. 2B. The results indicated that rs4823921 was still significantly related to the overall survival rate of gastric cancer patients and the prognosis of patients with genotype AC or AA were poor (HR 9.12 (1.240−67.01), *P* = 0.0299).

## Discussion

In this study, we mainly explored association of SNPs with GC susceptibility and prognosis in population in Wuwei, Gansu. We found that 31 SNPs were significantly related to the incidence of GC in Wuwei and rs4823921 could be well used to predict the OS of GC patients in Wuwei. Moreover, AC/AA genotype of rs4823921 polymorphism was significantly associated with the increased risk of GC in Wuwei population.

As we all know, the occurrence of GC is affected by lots of complex factors, including environmental factors, race, microbial infection, genetic background and so on [25, 26]. Numerous studies suggested that race and region are associated with the occurrence of GC. A study has showed that Asian/Pacific Islander males displayed the highest incidence and mortality when compared to other groups and genders [8]. Another study in Southern California found that ethnic minorities had a 40–50% increase in risk of GC compared with the non-Hispanic white population [20]. Moreover, there is relatively high incidence of GC in Asia and China [9–11].

Recently, increasing numbers of researches found that SNPs play an important role in the development of GC both in China and other countries [12, 17, 21]. A research in northeast China documented that four SNPs of TOB1 gene play an important role in the occurrence and development of GC in the Chinese Han population [27]. Not only in GC, polymorphism has also been documented to correlate with some other cancers’ susceptibility. In Caucasians, COX-2 rs689466 polymorphism was evidenced to associate with the colorectal cancer susceptibility [28]. Additionally, polymorphism might be potentially conducive to treatment choice for patients, and Wang et al. reported that the gefitinib-associated EGFR mutations were rarely occurred in adenocarcinoma of esophagogastric junction [29]. However, association of SNPs with GC susceptibility and prognosis in population in Wuwei has never been studied. Wuwei, Gansu, a city in northwest China, is one of the highest GC incidence’s area in China [12]. There are many possible reasons of the onset of GC, such as eating habits and environmental factors, but there are no researches related to genetic factors in this area. Consequently, the SNP chip analysis was conducted on 503 local participants in our present study. Based on the SNP analysis, 31 SNPs were significantly related to the incidence of GC in Wuwei. In addition, after multivariate Cox regression analysis, rs4823921 was still significantly related to the OS of GC patients and AC/AA genotype of rs4823921 polymorphism was significantly associated with an increased risk of GC in Wuwei population. Similarly, data from a case-control study suggested that the CT/TT genotype of DACT1 rs863091 polymorphism is associated with a decreased risk of GC in the Chinese Han population [30]. Chu et al. have demonstrated the CDH1 −160C→A promoter polymorphism and haplotypes affected the risk of sporadic diffuse GC in a Taiwanese population [31]. However, to the best of our knowledge, we have firstly identified a GC susceptibility related SNP in Wuwei. After searching the literature, we noticed that rs4823921 polymorphism has never been reported in any cancer but it was significantly related to the incidence of GC in Wuwei, which may explain the particularity of GC susceptibility in Wuwei area. Subsequently, rs4823921 was subjected to the annotation analysis and we found it was located on the downstream of LINC01310. A recent report in the Chinese Han population has demonstrated the serum mineral elements related susceptibility genes, among which LINC01310 was found to associated with the serum copper (Cu) concentration [32]. The increased GC risk was observed with growing level of serum Cu [33]. Another study has documented positive association between serum Cu and GC [34], which supported our results indirectly. Collectively, the GC susceptibility related SNP we identified might be meaningful in Wuwei, which deserves further exploration in near future.

Although our findings provided more reference information regarding the Wuwei regional GC susceptibility study, there were still several disadvantages in our research. Firstly, the location of crucial SNP rs4823921 partly limited the mining of its role, which should be further explored and illustrated combining multiple data type in the future. Moreover, the larger sample size is able to improve the reliability of our findings.

## Conclusions

In conclusion, via SNP chip, 31 SNPs were identified to be significantly related to the incidence of GC in Wuwei area and one genotype rs4823921 could probably predict the OS of GC in Wuwei area, which may contribute to further understand the complex reasons for relatively high incidence of GC in Wuwei area. Further exploration is still needed to clarify the underlying mechanisms.

## Supplementary Information

**Additional file 1: Table S1**. The detailed histopathology and pathological TNM information of all samples.

**Additional file 2: Table S2.** Alleles distribution of SNPs significantly associated with gastric cancer risk.

**Additional file 3: Table S3.** Genotype distribution of SNPs significantly related to gastric cancer risk.

**Additional file 4: Table S4.** Univariate Cox regression analysis.

## Data Availability

The datasets used and analyzed in the present research are available from the corresponding author on reasonable request.

## Abbreviations

GC: Gastric cancer
SNP: Single nucleotide polymorphisms
LD: Linkage disequilibrium
IBS: Identity by state
PC: Principal component analysis
OS: Overall survival
