# Supplementary material for: Association of single nucleotide polymorphisms (SNPs) with gastric cancer susceptibility and prognosis in population in Wuwei, Gansu, China
Source: World J Surg Oncol. 2022 Jun 11;20:194. doi: 10.1186/s12957-022-02663-6 (PMC9188220; doi:10.1186/s12957-022-02663-6)
Supplement: Supplementary file 2 — Additional file 2: Table S2. Alleles distribution of SNPs significantly associated with gastric cancer risk. [file 12957_2022_2663_MOESM2_ESM.docx]

Table S2 Alleles distribution of SNPs significantly associated with gastric cancer risk

| SNP ID | SNP Position | Gene | Region | Allele | Case | | Control | | PFDR | OR(95%CI) |
| --- | --- | --- | --- | --- | --- | --- | --- | --- | --- | --- |
|  |  |  |  |  | N | % | N | % |  |  |
| rs77938938 | chr10:8782674 | LOC105376398,LINC02676 | intergenic | T | 294 | 51.04 | 31 | 8.61 | 3.07E-35 | 11.06(7.39-16.55) |
|  |  |  |  | C | 282 | 48.96 | 329 | 91.39 |  |  |
| rs7640543 | chr3:30420911 | RBMS3,LINC01985 | intergenic | A | 299 | 0.11 | 53.78 | 40.00 | 3.89E-31 | 9.54(6.60-13.79) |
|  |  |  |  | G | 257 | 46.22 | 328 | 89.13 |  |  |
| rs76903750 | chr12:97720909 | LOC643711 | ncRNA_intronic | C | 100 | 18.12 | 8 | 2.08 | 9.03E-06 | 10.4(4.99-21.64) |
|  |  |  |  | T | 452 | 81.88 | 376 | 97.92 |  |  |
| rs28651066 | chr17:20806428 | LOC100287072,CCDC144NL | intergenic | G | 93 | 16.85 | 10 | 2.59 | 1.86E-05 | 7.62(3.91-14.83) |
|  |  |  |  | A | 459 | 83.15 | 376 | 97.41 |  |  |
| rs74362389 | chr12:42588999 | PRICKLE1 | intronic | C | 99 | 17.93 | 14 | 3.65 | 2.58E-05 | 5.78(3.24-10.28) |
|  |  |  |  | T | 453 | 82.07 | 370 | 96.35 |  |  |
| rs138286907 | chr17:28962210 | SEZ6 | intronic | G | 98 | 18.08 | 13 | 3.39 | 2.58E-05 | 6.30(3.47-11.42) |
|  |  |  |  | A | 444 | 81.92 | 371 | 96.62 |  |  |
| rs200612063 | chr2:203440845 | RAPH1 | exonic | G | 86 | 16.10 | 6 | 1.58 | 4.60E-05 | 11.97(5.17-27.69) |
|  |  |  |  | A | 448 | 83.90 | 374 | 98.42 |  |  |
| rs77837731 | chr8:68527938 | C8orf34 | intronic | C | 96 | 17.58 | 5 | 1.30 | 8.73E-05 | 16.26(6.54-40.36) |
|  |  |  |  | T | 450 | 82.42 | 381 | 98.71 |  |  |
| rs654638 | chr11:65849853 | SNX32 | intronic | G | 82 | 15.24 | 5 | 1.31 | 1.12E-04 | 13.56(5.44-33.79) |
|  |  |  |  | A | 456 | 84.76 | 377 | 98.69 |  |  |
| rs71398298 | chr15:67141933 | SMAD3 | intronic | C | 82 | 14.96 | 10 | 2.59 | 2.01E-04 | 6.62(3.38-12.94) |
|  |  |  |  | T | 466 | 85.04 | 376 | 97.41 |  |  |
| rs28698945 | chr4:8850597 | HMX1 | intronic | G | 111 | 20.48 | 26 | 6.81 | 4.38E-04 | 3.53(2.25-5.528) |
|  |  |  |  | A | 431 | 79.52 | 356 | 93.19 |  |  |
| rs12355139 | chr10:20276836 | PLXDC2 | intronic | G | 83 | 15.37 | 3 | 0.79 | 5.89E-04 | 22.94(7.19-73.18) |
|  |  |  |  | T | 457 | 84.63 | 379 | 99.21 |  |  |
| rs1894211 | chr11:75500151 | GDPD5 | intronic | G | 106 | 19.70 | 26 | 6.77 | 5.89E-04 | 3.38(2.15-5.31) |
|  |  |  |  | A | 432 | 80.30 | 358 | 93.23 |  |  |
| rs10781306 | chr9:68976477 | PIP5K1B | intronic | G | 80 | 14.87 | 9 | 2.33 | 6.19E-04 | 7.32(3.62-14.77) |
|  |  |  |  | A | 458 | 85.13 | 377 | 97.67 |  |  |
| rs7842319 | chr8:94640758 | ESRP1 | upstream | G | 109 | 20.19 | 25 | 6.55 | 1.92E-03 | 3.61(2.287-5.70) |
|  |  |  |  | T | 431 | 79.81 | 357 | 93.46 |  |  |
| rs3853818 | chr17:7442983 | FGF11 | intronic | C | 114 | 20.65 | 28 | 7.41 | 1.96E-03 | 3.28(2.10-5.03) |
|  |  |  |  | T | 438 | 79.35 | 350 | 92.59 |  |  |
| rs117937228 | chr20:32003199 | XKR7 | UTR3 | G | 82 | 15.19 | 14 | 3.65 | 2.03E-03 | 4.73(2.64-8.47) |
|  |  |  |  | T | 458 | 84.81 | 370 | 96.35 |  |  |
| rs141620966 | chr3:27315885 | NEK10 | intronic | C | 86 | 16.10 | 2 | 0.52 | 2.26E-03 | 36.67(8.96-150) |
|  |  |  |  | T | 448 | 83.90 | 382 | 99.48 |  |  |
| rs17643100 | chr13:32978544 | LINC00423,KL | intergenic | G | 151 | 28.38 | 52 | 13.54 | 5.35E-03 | 2.53(1.78-3.58) |
|  |  |  |  | A | 381 | 71.62 | 332 | 86.46 |  |  |
| rs146971769 | chr4:102980615 | SLC9B1 | intronic | C | 82 | 15.02 | 2 | 0.54 | 5.55E-03 | 32.69(7.98-133.8) |
|  |  |  |  | A | 464 | 84.98 | 370 | 99.46 |  |  |
| rs12986087 | chr19:8248434 | CERS4 | intronic | C | 81 | 14.67 | 2 | 0.53 | 6.20E-03 | 32.16(7.85-131.7) |
|  |  |  |  | A | 471 | 85.33 | 374 | 99.47 |  |  |
| rs786906 | chr1:88805891 | PKN2 | exonic | C | 214 | 37.68 | 201 | 52.07 | 7.01E-03 | 0.56(0.42-0.723) |
|  |  |  |  | T | 354 | 62.32 | 185 | 47.93 |  |  |
| rs78326603 | chr5:135862851 | SLC25A48 | intronic | C | 76 | 13.82 | 2 | 0.53 | 9.74E-03 | 30.3(7.39-124.2) |
|  |  |  |  | T | 474 | 86.18 | 378 | 99.47 |  |  |
| rs8107107 | chr19:41973158 | ATP1A3 | intronic | C | 118 | 22.01 | 38 | 10.00 | 1.01E-02 | 2.54(1.71-3.76) |
|  |  |  |  | T | 418 | 77.99 | 342 | 90.00 |  |  |
| rs421490 | chr1:8215224 | LINC01714 | downstream | G | 137 | 25.00 | 47 | 12.30 | 1.21E-02 | 2.38(1.65-3.41) |
|  |  |  |  | A | 411 | 75.00 | 335 | 87.70 |  |  |
| rs7263718 | chr20:381637 | TRIB3 | intronic | G | 118 | 22.18 | 37 | 9.64 | 1.21E-02 | 2.67(1.79-3.97) |
|  |  |  |  | A | 414 | 77.82 | 347 | 90.37 |  |  |
| rs4823921 | chr22:49161377 | LINC01310,NONE | intergenic | C | 213 | 39.44 | 90 | 23.32 | 1.35E-02 | 2.14(1.59-2.87) |
|  |  |  |  | A | 327 | 60.56 | 296 | 76.68 |  |  |
| rs3826729 | chr19:17247324 | NR2F6,USHBP1 | intergenic | G | 190 | 33.93 | 75 | 19.53 | 2.26E-02 | 2.12(1.55-2.87) |
|  |  |  |  | A | 370 | 66.07 | 309 | 80.47 |  |  |
| rs10487285 | chr7:106759502 | CCDC71L,PIK3CG | intergenic | G | 278 | 48.26 | 127 | 32.90 | 4.02E-02 | 1.90(1.45-2.48) |
|  |  |  |  | A | 298 | 51.74 | 259 | 67.10 |  |  |
| rs713383 | chr6:42272631 | TRERF1 | intronic | A | 179 | 31.63 | 177 | 46.09 | 4.98E-02 | 0.54(0.413-0.71) |
|  |  |  |  | G | 387 | 68.37 | 207 | 53.91 |  |  |
| rs9463078 | chr6:45213603 | SUPT3H | intronic | G | 174 | 31.64 | 170 | 45.45 | 4.98E-02 | 0.56(0.423-0.73) |
|  |  |  |  | A | 376 | 68.36 | 204 | 54.55 |  |  |
