# Supplementary material for: Association of single nucleotide polymorphisms (SNPs) with gastric cancer susceptibility and prognosis in population in Wuwei, Gansu, China
Source: World J Surg Oncol. 2022 Jun 11;20:194. doi: 10.1186/s12957-022-02663-6 (PMC9188220; doi:10.1186/s12957-022-02663-6)
Supplement: Supplementary file 3 — Additional file 3: Table S3. Genotype distribution of SNPs significantly related to gastric cancer risk. [file 12957_2022_2663_MOESM3_ESM.docx]

Table S3 Genotype distribution of SNPs significantly related to gastric cancer risk

| SNP | Allele | Group | AA | AB | BB | P |
| --- | --- | --- | --- | --- | --- | --- |
| rs421490 | G/A | Case | 8 | 121 | 145 | 1.24E-07 |
|  |  | Control | 4 | 39 | 148 |  |
| rs786906 | C/T | Case | 2 | 210 | 72 | 2.10E-22 |
|  |  | Control | 56 | 89 | 48 |  |
| rs200612063 | G/A | Case | 0 | 86 | 181 | 2.09E-18 |
|  |  | Control | 1 | 4 | 185 |  |
| rs141620966 | C/T | Case | 0 | 86 | 181 | 6.88E-21 |
|  |  | Control | 0 | 2 | 190 |  |
| rs7640543 | A/G | Case | 35 | 229 | 14 | 8.55E-66 |
|  |  | Control | 1 | 38 | 145 |  |
| rs28698945 | G/A | Case | 7 | 97 | 167 | 3.13E-09 |
|  |  | Control | 0 | 26 | 165 |  |
| rs146971769 | C/A | Case | 0 | 82 | 191 | 1.36E-18 |
|  |  | Control | 0 | 2 | 184 |  |
| rs78326603 | C/T | Case | 0 | 76 | 199 | 2.69E-17 |
|  |  | Control | 0 | 2 | 188 |  |
| rs713383 | A/G | Case | 25 | 129 | 129 | 1.78E-05 |
|  |  | Control | 36 | 105 | 51 |  |
| rs9463078 | G/A | Case | 3 | 168 | 104 | 3.54E-15 |
|  |  | Control | 44 | 82 | 61 |  |
| rs10487285 | G/A | Case | 68 | 142 | 78 | 1.19E-05 |
|  |  | Control | 19 | 89 | 85 |  |
| rs77837731 | C/T | Case | 8 | 80 | 185 | 2.88E-17 |
|  |  | Control | 0 | 5 | 188 |  |
| rs7842319 | G/T | Case | 11 | 87 | 172 | 7.89E-09 |
|  |  | Control | 2 | 21 | 168 |  |
| rs10781306 | G/A | Case | 4 | 72 | 193 | 2.51E-12 |
|  |  | Control | 1 | 7 | 185 |  |
| rs77938938 | T/C | Case | 21 | 252 | 15 | 4.71E-80 |
|  |  | Control | 7 | 17 | 156 |  |
| rs12355139 | G/T | Case | 0 | 83 | 187 | 1.86E-18 |
|  |  | Control | 0 | 3 | 188 |  |
| rs654638 | G/A | Case | 0 | 82 | 187 | 4.15E-16 |
|  |  | Control | 0 | 5 | 186 |  |
| rs1894211 | G/A | Case | 3 | 100 | 166 | 2.06E-09 |
|  |  | Control | 1 | 24 | 167 |  |
| rs74362389 | C/T | Case | 3 | 93 | 180 | 1.10E-12 |
|  |  | Control | 0 | 14 | 178 |  |
| rs76903750 | C/T | Case | 4 | 92 | 180 | 1.12E-16 |
|  |  | Control | 0 | 8 | 184 |  |
| rs17643100 | G/A | Case | 22 | 107 | 137 | 5.64E-07 |
|  |  | Control | 3 | 46 | 143 |  |
| rs71398298 | C/T | Case | 1 | 80 | 193 | 9.31E-12 |
|  |  | Control | 0 | 10 | 183 |  |
| rs3853818 | C/T | Case | 10 | 94 | 172 | 3.33E-08 |
|  |  | Control | 0 | 28 | 161 |  |
| rs28651066 | G/A | Case | 1 | 91 | 184 | 1.92E-14 |
|  |  | Control | 0 | 10 | 183 |  |
| rs138286907 | G/A | Case | 3 | 92 | 176 | 1.83E-15 |
|  |  | Control | 2 | 9 | 181 |  |
| rs12986087 | C/A | Case | 0 | 81 | 195 | 1.72E-18 |
|  |  | Control | 0 | 2 | 186 |  |
| rs3826729 | G/A | Case | 30 | 130 | 120 | 5.94E-06 |
|  |  | Control | 7 | 61 | 124 |  |
| rs8107107 | C/T | Case | 5 | 108 | 155 | 6.73E-07 |
|  |  | Control | 1 | 36 | 153 |  |
| rs7263718 | G/A | Case | 10 | 98 | 158 | 7.88E-07 |
|  |  | Control | 2 | 33 | 157 |  |
| rs117937228 | G/T | Case | 4 | 74 | 192 | 5.75E-09 |
|  |  | Control | 0 | 14 | 178 |  |
| rs4823921 | C/A | Case | 46 | 121 | 103 | 3.60E-06 |
|  |  | Control | 12 | 66 | 115 |  |
