# Supplementary material for: Association of single nucleotide polymorphisms (SNPs) with gastric cancer susceptibility and prognosis in population in Wuwei, Gansu, China
Source: World J Surg Oncol. 2022 Jun 11;20:194. doi: 10.1186/s12957-022-02663-6 (PMC9188220; doi:10.1186/s12957-022-02663-6)
Supplement: Supplementary file 4 — Additional file 4: Table S4. Univariate Cox regression analysis. [file 12957_2022_2663_MOESM4_ESM.docx]

Table S4 Univariate Cox regression analysis

| SNP |  | HR | HR.lower | HR.upper | HR (95% CI) | p.value |
| --- | --- | --- | --- | --- | --- | --- |
| Age |  | 1 | 0.98 | 1.1 | 1 (0.98-1.1) | 0.23 |
| Gender | Female |  |  |  | Reference |  |
|  | Male | 2.3 | 0.96 | 5.6 | 2.3 (0.96-5.6) | 0.061 |
| Sample Type | Sporadic |  |  |  | Reference |  |
|  | Heredofamilial | 1.1 | 0.53 | 2.1 | 1.1 (0.53-2.1) | 0.88 |
| rs10487285 | GG |  |  |  | Reference |  |
|  | AG+AA | 1.1 | 0.51 | 2.2 | 1.1 (0.51-2.2) | 0.88 |
| rs138286907 | AA |  |  |  | Reference |  |
|  | AG+GG | 0.79 | 0.39 | 1.6 | 0.79 (0.39-1.6) | 0.49 |
| rs28698945 | AA |  |  |  | Reference |  |
|  | AG+GG | 0.66 | 0.33 | 1.4 | 0.66 (0.33-1.4) | 0.26 |
| rs17643100 | GG |  |  |  | Reference |  |
|  | AA+AG | 0.61 | 0.21 | 1.8 | 0.61 (0.21-1.8) | 0.36 |
| rs713383 | AA |  |  |  | Reference |  |
|  | AG+GG | 0.94 | 0.33 | 2.7 | 0.94 (0.33-2.7) | 0.9 |
| rs786906 | TT |  |  |  | Reference |  |
|  | TC+CC | 0.92 | 0.45 | 1.9 | 0.92 (0.45-1.9) | 0.81 |
| rs8107107 | TT |  |  |  | Reference |  |
|  | TC+CC | 0.63 | 0.31 | 1.3 | 0.63 (0.31-1.3) | 0.19 |
| rs9463078 | AA |  |  |  | Reference |  |
|  | AG+GG | 0.86 | 0.44 | 1.7 | 0.86 (0.44-1.7) | 0.67 |
| rs12355139 | TT |  |  |  | Reference |  |
|  | TG+GG | 1.8 | 0.9 | 3.7 | 1.8 (0.9-3.7) | 0.093 |
| rs71398298 | TT |  |  |  | Reference |  |
|  | TC+CC | 1.6 | 0.78 | 3.4 | 1.6 (0.78-3.4) | 0.19 |
| rs141620966 | TT |  |  |  | Reference |  |
|  | TC+CC | 1.5 | 0.72 | 3.1 | 1.5 (0.72-3.1) | 0.27 |
| rs77837731 | TT |  |  |  | Reference |  |
|  | TC+CC | 0.58 | 0.28 | 1.2 | 0.58 (0.28-1.2) | 0.14 |
| rs10781306 | AA |  |  |  | Reference |  |
|  | AG+GG | 0.55 | 0.26 | 1.1 | 0.55 (0.26-1.1) | 0.11 |
| rs654638 | AA |  |  |  | Reference |  |
|  | AG+GG | 0.64 | 0.31 | 1.3 | 0.64 (0.31-1.3) | 0.23 |
| rs76903750 | TT |  |  |  | Reference |  |
|  | TC+CC | 0.61 | 0.3 | 1.2 | 0.61 (0.3-1.2) | 0.16 |
| rs77938938 | TT |  |  |  | Reference |  |
|  | TC+CC | 0.85 | 0.2 | 3.6 | 0.85 (0.2-3.6) | 0.83 |
| rs28651066 | AA |  |  |  | Reference |  |
|  | AG+GG | 0.74 | 0.37 | 1.5 | 0.74 (0.37-1.5) | 0.4 |
| rs7263718 | GG |  |  |  | Reference |  |
|  | AG+AA | 1 | 0.31 | 3.4 | 1 (0.31-3.4) | 0.98 |
| rs78326603 | TT |  |  |  | Reference |  |
|  | TC+CC | 1.7 | 0.78 | 3.5 | 1.7 (0.78-3.5) | 0.19 |
| rs74362389 | TT |  |  |  | Reference |  |
|  | TC+CC | 0.7 | 0.35 | 1.4 | 0.7 (0.35-1.4) | 0.31 |
| rs1894211 | AA |  |  |  | Reference |  |
|  | AG+GG | 0.72 | 0.36 | 1.4 | 0.72 (0.36-1.4) | 0.35 |
| rs3853818 | TT |  |  |  | Reference |  |
|  | TC+CC | 0.51 | 0.25 | 1 | 0.51 (0.25-1) | 0.054 |
| rs421490 | GG |  |  |  | Reference |  |
|  | AG+AA | 0.96 | 0.23 | 4 | 0.96 (0.23-4) | 0.96 |
| rs3826729 | GG |  |  |  | Reference |  |
|  | AG+AA | 0.76 | 0.29 | 2 | 0.76 (0.29-2) | 0.57 |
| rs12986087 | AA |  |  |  | Reference |  |
|  | AC+CC | 0.79 | 0.39 | 1.6 | 0.79 (0.39-1.6) | 0.51 |
| rs7842319 | TT |  |  |  | Reference |  |
|  | TG+GG | 0.69 | 0.35 | 1.3 | 0.69 (0.35-1.3) | 0.27 |
| rs117937228 | TT |  |  |  | Reference |  |
|  | TG+GG | 0.68 | 0.33 | 1.4 | 0.68 (0.33-1.4) | 0.29 |
| rs200612063 | AA |  |  |  | Reference |  |
|  | AA+AG | 0.72 | 0.36 | 1.4 | 0.72 (0.36-1.4) | 0.35 |
| rs4823921 | CC |  |  |  | Reference |  |
|  | AC+AA | 9.3 | 1.3 | 68 | 9.3 (1.3-68) | 0.028 |
| rs7640543 | AA |  |  |  | Reference |  |
|  | AG+GG | 1.1 | 0.31 | 3.6 | 1.1 (0.31-3.6) | 0.94 |
